# Self-Doped Conjugated Polymeric Binders Improve the Capacity and Mechanical Properties of V_2_O_5_ Cathodes

**DOI:** 10.3390/polym11040589

**Published:** 2019-04-01

**Authors:** Xiaoyi Li, Hyosung An, Joseph Strzalka, Jodie Lutkenhaus, Rafael Verduzco

**Affiliations:** 1 Department of Chemical and Biomolecular Engineering, Rice University, Houston, TX 77005, USA; xl44@rice.edu; 2 Artie McFerrin Department of Chemical Engineering, Texas A&M University, College Station, TX 77843, USA; qcan17pp@tamu.edu; 3 X-ray Science Division, Argonne National Laboratory, Lemont, IL 60439, USA; strzalka@anl.gov; 4 Department of Materials Science and Engineering, Texas A&M University, College Station, TX 77843, USA; 5 Department of Materials Science and NanoEngineering, Rice University, Houston, TX 77005, USA

**Keywords:** conjugated polymer, self-doped polymer, lithium-ion battery, conductive binder, vanadium pentoxide

## Abstract

Polymeric binders serve to stabilize the morphology of electrodes by providing adhesion and binding between the various components. Successful binders must serve multiple functions simultaneously, including providing strong adhesion, improving conductivity, and providing electrochemical stability. A tradeoff between mechanical integrity and electrochemical performance in binders for lithium-ion batteries is one of the many challenges of improving capacity and performance. In this paper, we demonstrate a self-doped conjugated polymer, poly(9,9-bis(4′-sulfonatobutyl)fluorene-alt-co-1,4-phenylene) (PFP), which not only provides mechanical robustness but also improves electrode stability at temperatures as high as 450 °C. The self-doped PFP polymer is comprised of a conjugated polyfluorene backbone with sulfonate terminated side-chains that serve to dope the conjugated polymer backbone, resulting in stable conductivity. Composite electrodes are prepared by blending PFP with V_2_O_5_ in water, followed by casting and drying. Structural characterization with X-ray diffraction and wide-angle X-ray scattering shows that PFP suppresses the crystallization of V_2_O_5_ at high temperatures (up to 450 °C), resulting in improved electrode stability during cycling and improved rate performance. This study demonstrates the potential of self-doped conjugated polymers for use as polymeric binders to enhance mechanical, structural, and electrochemical properties.

## 1. Introduction

The widespread use and rapidly-growing demand of lithium-ion batteries has raised many researchers’ interests in further improving the lithium storage capacity and its long-term stability and safety [1,2,3,4,5,6,7]. A necessity in stable lithium-ion batteries is a binder to hold together the various components of the electrode and provide structural stability [8,9,10]. Most commonly used binders, such as poly(vinylidene fluoride) (PVDF), poly(tetrafluoroethylene) (PTFE), and carboxy methyl cellulose (CMC), are inactive in terms of ionic or electronic conductivity or electrochemical activity. As such, these binders are added in the lowest possible quantities (typically less than 5 wt % in cathodes) in order to maintain essential mechanical integrity without significantly sacrificing battery capacity. The tradeoff between electrochemical performance and mechanical properties can be potentially overcome by using a multifunctional binder, which provides not only mechanical robustness, but electronic conductivity as well.

Conjugated polymers that are doped can provide good intrinsic electronic conductivity while also enhancing mechanical properties. For example, the commercially available conductive polymer poly(3,4-ethylenedioxythiophene):poly(styrenesulfonate) (PEDOT:PSS) has been widely studied in device applications, including as a functional binder in carbon black free LiFeO_4_ cathodes for Li-ion batteries, resulting in improved stability and comparable capacity with conventional electrodes [11]. PEDOT:PSS has also been used as a conductive additive and binder in silicon anodes [12,13]. PEDOT:PSS was shown to help form homogeneous and continuous conducting bridges within the composite Si anode, which results in improved cycling and rate performance [12]. 

However, the performance of PEDOT:PSS can change dramatically with processing conditions, including the choice of solvent, concentration, and the presence of other additives, and achieving significant conductivity often requires post-processing treatment [14,15]. Self-doped conjugated polymers contain pendant, covalently bound ionic groups that serve to dope the conjugated polymer backbone. The resulting materials are more stable with respect to electronic properties and do not require the addition of external dopants [16,17,18]. These conducting polymers are commonly used in biosensors, transistors, and rechargeable batteries [19,20,21]. As the active material in lithium-ion batteries, the sulfonic groups from self-doped polymers help limit the participation of anions during the charge compensation process, leading to higher specific capacity and stability [22,23,24]. They have been implemented as polymer electrolytes for solid-state batteries, showing improved lithium transference number and ionic conductivity [25], and are also typically soluble in water. 

In this study, we investigated a self-doped conjugated polymer as a binder in vanadium pentoxide (V_2_O_5_) cathodes. V_2_O_5_ is a promising cathode material with a high theoretical capacity of 443 mAhg^−1^ (assuming the intercalation of three lithium ions) and a specific energy of 1218 mWhg^−1^ (assuming a nominal 2.75 V discharge voltage) [26]. However, V_2_O_5_ lacks intrinsic conductivity, requiring the use of conductive additives along with a polymeric binder [27,28,29]. We focused on the self-doped polymer PFP (poly(9,9-bis(4′-sulfonatobutyl)fluorene-alt-co-1,4-phenylene)), which has previously been used as a work-function modifier for organic solar cells [30] and in thermoelectric devices [31]. Critically, the polymer is water-soluble and can be incorporated in V_2_O_5_ layers during aqueous processing. We incorporated PFP into V_2_O_5_ cathodes and characterized the binder’s impact on the structure, crystallinity, and electrochemical performance. Grazing-incidence X-ray scattering measurements and X-ray diffraction measurements showed that PFP suppressed the transition of layered V_2_O_5_ to the a-crystal phase, resulting in improved stability and performance. This study demonstrates the potential of self-doped conjugated polymers for use as polymeric binders to enhance mechanical, structural, and electrochemical properties.

## 2. Materials and Methods 

### 2.1. Materials

All of the reagents and solvents used were purchased commercially from Sigma-Aldrich (St. Louis, MO, USA) and VWR (Radnor, PA, USA), and they were used as received unless noted otherwise. Lithium ribbon was purchased from Alfa Aesar (Haverhill, MA, USA). All coin cell-related products including CR2032 coin cell cases, springs, and stainless steel coins (15.8 mm diameter × 0.5 mm thickness) were purchased from MTI Corporation (Richmond, CA, USA).

### 2.2. Synthesis of V_2_O_5_ Xerogel

V_2_O_5_ xerogel was synthesized using a slightly modified version of the procedure reported by Liu et al. [26]. A solution of sodium metavanadate (0.1 M) in DI water was passed through an ion-exchange column filled with (Dowex-50-WX2, 50–100 mesh). The column was washed with Millipore water until the eluent appeared colorless and had a neutral pH value. The eluent (HVO_3_ in water) was collected and aged for 3 weeks at room temperature to produce a dark red V_2_O_5_ dispersion. The final homogeneous V_2_O_5_ xerogel solid was obtained by freeze-drying the aged solution at low a temperature and under vacuum.

### 2.3. Synthesis of Poly(9,9-bis(4′-sulfonatobutyl)fluorene-alt-co-1,4-phenylene) (PFP)

In a 10 mL microwave reaction tube, benzene-1,4-diboronic acid (100 mg) and 2,7-dibromo-9,9-bis(40-sulfonatobutyl)fluorene (157 mg) were added, followed by 3 mL of DMF solvent and 1.2 mL of 2 M aqueous sodium carbonate. Upon the addition of 1–2 drops of Aliquat 366, the mixture was stirred while purging through N_2_ for 15 min. The catalyst (PPh_3_)_4_Pd(0) (34 mg) was then added quickly to the mixture, and the reaction vial was sealed. The mixture was allowed to react via microwave at 160 °C for 1 h. The raw product was obtained through precipitation in acetone. Precipitate was collected by filtration and washed with a large amount of acetone and methanol until no obvious color could be seen in the filtrate. The precipitate was then completely dissolved in 25 mL of Millipore water and transferred to a dialysis bag (MWCO: ~5000). The dialysis bag was placed in a 1 L beaker filled with water and stirred for 3 days; the water inside the beaker was changed every 12 h. After dialysis, the final purified product was recovered from water through freeze-drying.

### 2.4. Preparation of Electrodes

Before use, the stainless steel spacers were washed thoroughly by sonication in soap water, followed by DI water, IPA, and acetone, each for 15 min. All electrode solutions were prepared in Millipore water. V_2_O_5_ and the PFP polymer were first dissolved in water separately and sonicated to provide a uniform solution. Then the two solutions were mixed in different ratios to obtain a series of 0–20 wt % PFP with V_2_O_5_ solution. The hybrid electrode solution was drop-cast on stainless steel coins, targeting a dried solid mass of ~1 mg. After preliminary drying under a fume hood, the resulting electrodes were transferred into a nitrogen-filled glove box to be heated at 90 °C overnight, in order to further remove residual water.

Thermally annealed samples were prepared in a similar fashion. After drying at 90 °C overnight, the sample was annealed at a certain temperature (ranging from 150 °C to 450 °C) under N_2_ for 30 min. The sample was always placed under a N_2_ atmosphere after annealing in order to prevent the re-absorption of water in air, until it was removed for testing purposes.

### 2.5. Coin Cell Assembly and Testing

Coin cells were assembled inside a water-free, oxygen-free Ar glove box, and pure lithium metal was used as the anode. The electrolyte used was 1M LiTFSI in propylene carbonate, and the separator was Celgard 3501 (Celgard LLC, Charlotte, NC, USA). All coin cells were tested for cyclic voltammetry and galvanostatic charge-discharge measurements, with an Arbin Battery Testing Instrument (Arbin Instruments, College Station, TX, USA). 

### 2.6. Structural Characterization

X-ray spectroscopy characterization was carried out by a Rigaku Ultima II vertical powder diffractometer (Rigaku Corporation, The Woodlands, TX, USA) using Cu K-alpha radiation (λ = 1.5418 Å) with Bragg–Brentano para-focusing optics at 40 kV. All X-ray diffraction (XRD) samples were prepared on cover glass to eliminate any background noise from substrate. Sample solutions were drop-cast onto the cover glass and dried in a vacuum chamber. Thermal annealing was conducted under a N_2_ atmosphere.

Grazing-incidence X-ray-scattering in a wide-angle regime (GIWAXS) was performed on the beam line 8-ID-E at the Advanced Photon Source, Argonne National Laboratory [32]. A monochromator with a Si (111) single crystal was used to provide an X-ray beam of 7.35 keV. All samples were prepared on a 1” × 1” silicon wafer via drop-casting, then annealed under nitrogen for 30 min. The test was carried out at room temperature under a vacuum.

### 2.7. Mechanical Measurements

Samples for the peel test were prepared in the exact same way as cell electrodes. 3M Scotch tape was applied to the whole surface of each sample, flattened, and then gradually peeled off. The amount of electrode remaining adhered to the substrate was determined visually.

Dynamic mechanical analysis (DMA) failure test was conducted using an ARES G2 rheometer. Free-standing films of various PFP/V_2_O_5_ blends were prepared by drop-casting on a small plastic weigh boat and peeling off the film after drying in the fume hood. The film was then cut into approximately 10 mm high by 3 mm wide segments and both ends were fixed by Kapton tape. A constant strain rate (0.001 mm/s) was applied, and the sample was stretched until failure.

## 3. Results

### 3.1. Structual Analysis with Thermal Annealing

**Figure 1:**
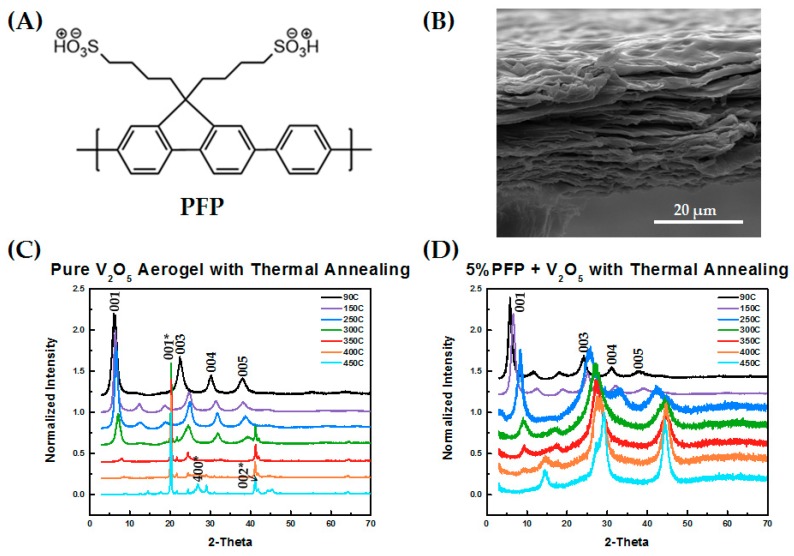
(**A**) Chemical structure of poly(9,9-bis(4′-sulfonatobutyl)fluorene-alt-co-1,4-phenylene) (PFP); (**B**) SEM image of V_2_O_5_ layered structure; (**C**,**D**) X-ray diffraction (XRD) patterns with thermal annealing from 90 °C to 450 °C for (**C**) pure V_2_O_5_ and (**D**) V_2_O_5_ blends with 5 wt % PFP, respectively.

The structure of the self-doped polymer PFP is shown in Figure 1A along with an SEM micrograph of the V_2_O_5_ xerogel’s layered structure. The layered structure with water molecules as “pillars” between V_2_O_5_ sheets provides a high storage capacity for lithium, and our first goal was to determine the impact that the self-doped polymer PFP had on the layered structure. X-ray measurements were used to quantify the structure and periodicity of the V_2_O_5_ xerogel. 

We conducted X-ray diffraction measurements to characterize the structure of V_2_O_5_ with thermal annealing as well as with and without the PFP polymeric binder. Thermal annealing is critical for improving the storage capacity of V_2_O_5_, since water molecules within the V_2_O_5_ layered structure can react with lithium to form Li_2_O, resulting in a poor cycle life without annealing [33,34]. However, excessive annealing can lead to the collapse of the layered structure [26] and the crystallization of V_2_O_5_ to form the orthorhombic phase, which can only intercalate one Li ion while the xerogel phase can intercalate three equivalents [35]. The increased distance between adjacent layers in the xerogel phase also improves the diffusion of Li ions through the electrode, hence improving the overall Li ion capacity [36].

Figure 1C shows that prior to thermal annealing, the V_2_O_5_ had the expected layered structure characteristic of the xerogel phase, with a layer spacing of 1.47 nm, shown by the typical (001), (003), (004), and (005) diffraction peaks. With heating, the layered structure was maintained up to 300 °C with a noticeable decrease in the layered spacing, which was reflected in a shift of the primary diffraction peak to higher angles. At annealing temperatures at and above 350 °C, crystallization occurred, evidenced by a crystallization peak at 20°. Crystallization and complete transition to the orthorhombic phase was achieved at 400 °C and above, also corresponding to the indexed orthorhombic V_2_O_5_ structure (JCPDS card No 41-1426) [26,36,37].

The phase behavior of the V_2_O_5_ with added PFP polymer exhibited a very different trend with thermal annealing. Initially, prior to thermal annealing, a layered xerogel structure with (00l) peaks was observed, which is similar to that for pure V_2_O_5_ but with a slightly larger interlayer spacing of 1.60 nm. With heating, a larger shift in the primary peak position was observed, but the layered structure was maintained up to an annealing temperature of 450 °C. The relative intensities of the diffraction peaks change significantly with annealing, which may be related to the loss of water during annealing, affecting the scattering length density of the region between the layers, and/or a change in the contrast due to the decrease in layer size. Thermogravimetric analysis (TGA) measurements were conducted to verify that the materials and polymer are stable to annealing temperatures of up to 450 °C (see Supporting Information Figure S1). 

**Figure 2:**
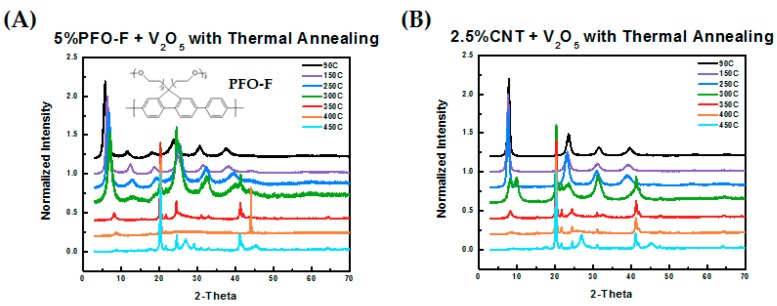
(**A**) Schematic of PFO-F and XRD pattern of V_2_O_5_ blended with 5% PFO-F undergoing thermal annealing from 90 °C to 450 °C. (**B**) XRD pattern of V_2_O_5_ blended with 2.5% carbon nanotubes (CNTs) undergoing thermal annealing from 90 °C to 450 °C.

As a control study, we examined the spectrum of another conjugated polymeric binder, PFO-F, which has a similar chemical structure to PFP except with uncharged, oligo-ethylene glycol side-chains. The molecular structure of PFO-F and XRD analysis of cathodes of PFO-F in V_2_O_5_ are provided in Figure 2A. This analysis shows that the crystallization of V_2_O_5_ occurred with the PFO-F additive just like that of pure V_2_O_5_. This reflects the poor or lack of intercalation of the PFO-F polymer between the V_2_O_5_ layers. Without the sulfonate groups, the PFO-F polymer had poorer water solubility and weaker interactions with V_2_O_5_. Furthermore, we also investigated the possible effect of adding carbon nanotubes (CNTs), which is a common non-polymeric additive to enhance electronic conductivity. As shown in Figure 2B, once again the crystallization of V_2_O_5_ was not suppressed. These experiments indicate that PFP stabilizes the xerogel structure of V_2_O_5_ at high annealing temperatures, up to 450 °C.

**Figure 3:**
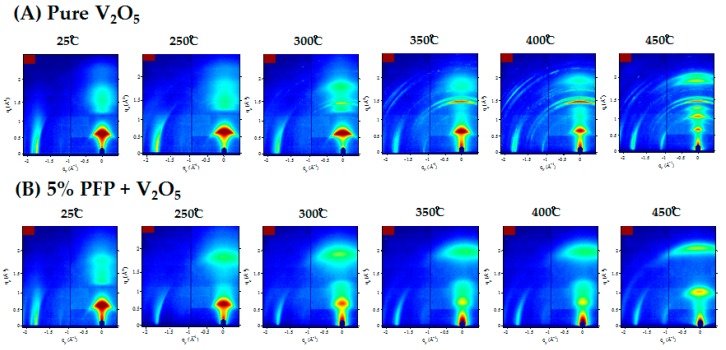
Grazing-incidence X-ray-scattering (GIWAXS) 2-D images with ex situ thermal annealing from 25 °C to 450 °C for (**A**) pure V_2_O_5_ and (**B**) V_2_O_5_ blended with 5% PFP.

To further investigate crystallization of V_2_O_5_ xerogel under thermal annealing, we conducted grazing-incidence wide angle X-ray (GIWAXS) measurements for all of the above annealed samples. In Figure 3A, we can clearly see that at 350 °C, multiple sharp diffraction peaks reflecting the crystallization of V_2_O_5_ appear, consistent with the powder XRD studies. When the temperature was increased to 400 °C and 450 °C, the complete crystallization of V_2_O_5_ occurred. All of the residual water molecules were removed from the V_2_O_5_ sheets and the layered structure collapsed. In Figure 3B, GIWAXS again confirmed that PFP stabilizes the layered structure at high temperatures. As discussed previously with the XRD patterns, initially the blend has a similar structure to pure V_2_O_5_ at room temperature. When the sample was heated, no multiple ring structure could be observed, up to 450 °C. We repeated the same procedure with the other two samples, which can be referred to in the Supporting Information (Figure S2). Neither of the V_2_O_5_/PFO-F or V_2_O_5_/CNT blends showed any signs of suppressing crystallization. The GIWAXS images once again confirmed our findings obtained through the XRD results in Figure 1.

### 3.2. Electrochemical Performance with Thermal Annealing

**Figure 4:**
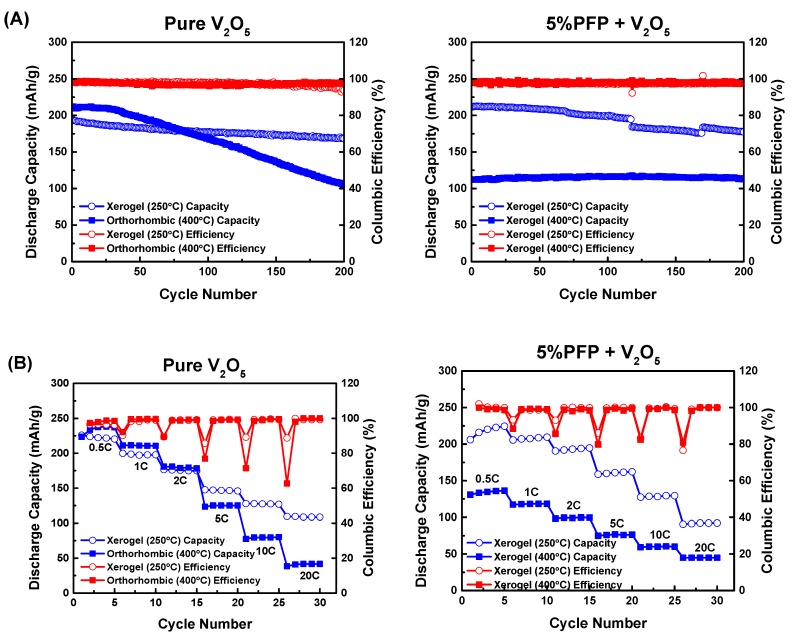
(**A**) Respective cycling performance of pure V_2_O_5_ and V_2_O_5_ + 5% PFP at 250 °C and 400 °C; (**B**) respective rate performance of pure V_2_O_5_ and V_2_O_5_ + 5% PFP at 250 °C and 400 °C. The small capacity drop for the 5% PFP sample between about 130 to 160 cycles is an artifact due to an unfortunate power interruption during testing.

We have demonstrated that self-doped polymer PFP as a binder helps to suppress the crystallization of V_2_O_5_ at high temperatures. Past research has shown that V_2_O_5_ crystallization is unfavorable towards battery behavior [34,36]; even though crystallized V_2_O_5_ electrodes have high initial capacity, their cycle life is severely damaged and their capacity degrades rapidly. We carried out electrochemical measurements for our own pure V_2_O_5_ and V_2_O_5_ + 5% PFP electrodes, and the results are shown below in Figure 4.

We evaluated energy storage performance through galvanostatic charge–discharge tests. All cells were analyzed for five cycles at 0.5 C rate, followed by five cycles at 1 C, 2 C, 5 C, 10 C, and 20 C rates. After rate performance tests, we cycled these cells at 1 C rate for 200 cycles. As a result, the pure V_2_O_5_ electrode annealed at 400 °C showed a slightly higher initial discharge capacity of ~210 mAh/g when compared with the V_2_O_5_ electrode before crystallization temperature (at 250 °C). However, its capacity started to drop rapidly after about 30 cycles and soon fell below the capacity of the V_2_O_5_ electrode annealed at 250 °C after 80 cycles. This rapid degradation is attributed to the crystallization of V_2_O_5_, which not only reduced the layer spacing for Li^+^ ion storage, but also compromised the mechanical robustness of the electrode, making it more vulnerable to irreversible structural change. For comparison, we also conducted galvanostatic charge-discharge tests on V_2_O_5_ electrodes with 10 wt % PVDF binder, and the results are provided in the Supporting Information Figure S5. The capacity of these electrodes is significantly lower than electrodes with PFP binder.

On the other hand, with the addition of 5% PFP, the hybrid electrode showed a marginally higher capacity at 250 °C than the pure V_2_O_5_ electrode. When the annealing temperature was raised past the usual crystallization temperature to 400 °C, although the PFP hybrid cell did not have a high initial capacity like the pure cell does, it was much more stable during cycling. The crystallization of V_2_O_5_ was suppressed in the presence of PFP, which reduced the capacity but improved the cycle life. Cyclic voltammetry (CV) scans revealed a single redox peak for 5% PFP at 400 °C, while the pure V_2_O_5_ electrodes showed two peaks when annealed at 400 °C (see Supporting Information Figure S6). Prior reports have observed that the second Li intercalation is less reversible [38,39], and our results are consistent with these prior studies. 

Similar to the rate performance tests, crystallized V_2_O_5_ electrodes displayed a slightly higher capacity at a slow charge–discharge rate; but the performance worsened quickly at higher C rates. Meanwhile, the 5% PFP hybrid electrode with crystallization suppression at 400 °C had a more stable pattern and showed less capacity loss from 0.5 C to 20 C.

### 3.3. Mechanical Analysis of PFP Hybrid Electrodes

**Figure 5:**
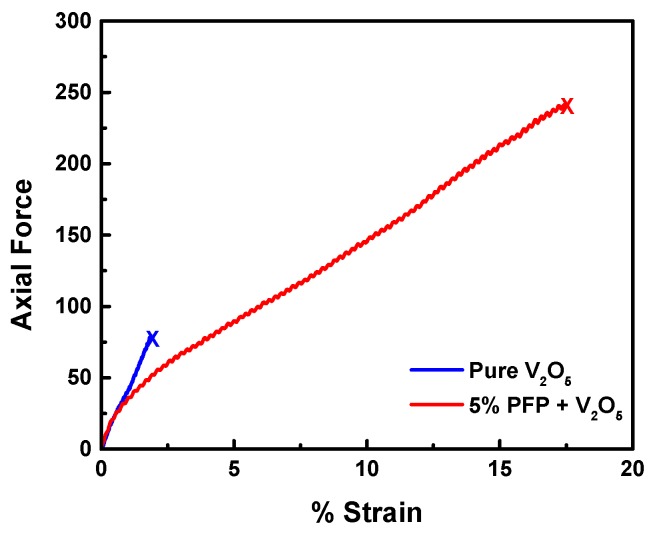
Dynamic mechanical analysis (DMA) failure tests for pure V_2_O_5_ and V_2_O_5_ with 5% PFP added.

DMA failure tests were conducted to examine the effect of PFP binder in the overall mechanical property of V_2_O_5_ hybrid electrodes. Samples of pure V_2_O_5_ and V_2_O_5_ + 5% PFP were stretched at a constant strain rate until failure. The resulting diagram is shown in Figure 5. As we can clearly see, the pure V_2_O_5_ electrodes had a very small % strain at failure of ~2%; while upon implementing even a small amount of PFP (5%), the blend was almost eight times more extensible than before. Therefore, PFP as a binder greatly enhances the general stretchability and durability of the hybrid electrode.

Finally, we evaluated the adhesion ability of these electrodes during thermal annealing with a series of peel tests. Images of samples annealed at various temperatures before and after peeling are listed in Figure S2. Generally speaking, as the annealing temperature increased, the adhesion of V_2_O_5_ worsened. Unfortunately, adding only 5 wt % of PFP as a binder in this case did not seem to improve this situation during thermal annealing. From Figure S3, we can see that at least 20 wt % of PFP needs to be added for a clean peel result, which is four times more than what we used in this study. However, we did not focus on PFP polymer content higher than 5 wt % due to the poor battery performance at higher binder contents. Figure S4 in the supporting information displays the galvanostatic charge–discharge cycling and various rate performances for 20 wt % PFP with V_2_O_5_. The energy storage capacity dropped to an extremely low level at higher binder contents. Therefore, although increasing the PFP binder content helps to enhance the adhesion of the material to the substrate, it also harms the electrochemical behavior of the hybrid electrode. This tradeoff between capacity and mechanical property should be taken into careful consideration. 

## 4. Discussion

In this work, we conducted a systematic study of a fully water-processable, thermally annealed V_2_O_5_ cathode with self-doped PFP polymer as the binder. The thermal annealing of V_2_O_5_ is favorable towards battery behavior up to the point when V_2_O_5_ starts to crystallize, around 300 °C. The crystallization of V_2_O_5_ at high temperatures disrupts the layered structure in the V_2_O_5_ xerogel, making it more vulnerable to permanent structural damage and thereby greatly reducing the cycle life of the battery. By adding only 5 wt % of PFP as a binder, we found that V_2_O_5_ crystallization is suppressed up to 450 °C. As a result, PFP improves the stability of V_2_O_5_ annealed at higher temperatures and helps to maintain desirable layer spacing within the V_2_O_5_ system for Li ion storage and diffusion. Cycling data has indicated that a 5% PFP/V_2_O_5_ hybrid electrode is stable for at least 200 cycles, even if it is annealed at 400 °C. 

In addition, PFP as a binder can also improve mechanical stretchability and reduce the stiffness of V_2_O_5_. Both properties contribute to a more stable electrode and longer cycle life during repeated charging and discharging. The use of 5 wt % PFP increased the hybrid electrode’s % strain at failure by around eight-fold. However, there are still some remaining problems with incorporating higher binder content in this system. Although a higher PFP content provides much better adhesion in a peel test, the electrochemical performance is largely diminished. We would need to carefully consider this tradeoff between battery performance and adhesion to the current collector.

Even though PEDOT:PSS has been a very popular research subject for its electrochemical behaviors, very little research has been carried out on other self-doped polymers used as binders. PFP is just one example of a synthesized self-doped polymer used as a binder in Li-ion batteries, and there is still a broad library of self-doped polymers beyond PEDOT:PSS that are of interest. These polymers have a good combination of electronic properties, hydrophilicity, solubility, etc. and therefore are good candidates for battery binders. Further research can be done to improve their conductivity, mechanical properties, electrolyte uptake, and more. 

## Supplementary Materials

The following are available online at https://www.mdpi.com/2073-4360/11/4/589/s1, Figure S1: Thermogravimetric analysis for PFP polymer and V_2_O_5_; Figure S2: GIWAXS 2-D images with ex situ thermal annealing from 25 °C to 450 °C for V_2_O_5_ blended with (A) 5% PFO-F and (B) 2.5% CNTs; Figure S3: Images of peel tests; Figure S4: Galvanostatic charge–discharge tests for V_2_O_5_ + 20%PFP; Figure S5: Galvanostatic charge–discharge tests for V_2_O_5_ + 10% PVDF + 10% Super-P Carbon; Figure S6: Cyclic voltammetry for (a) pure V_2_O_5_ and (b) 5% PFP + V_2_O_5_ annealed from 150 °C to 450 °C.

## References

[1] Scrosati B., Garche J. (2010). Lithium batteries: Status, prospects and future. J. Power Sources.

[2] Marom R., Amalraj S.F., Leifer N., Jacob D., Aurbach D. (2011). A review of advanced and practical lithium battery materials. J. Mater. Chem..

[3] Zhao W., Li X., Yin R., Qian L., Huang X., Liu H., Zhang J., Wang J., Ding T., Guo Z. (2019). Urchin-like NiO–NiCo_2_O_4_ heterostructure microsphere catalysts for enhanced rechargeable non-aqueous Li–O_2_ batteries. Nanoscale.

[4] Lou X., Lin C., Luo Q., Zhao J., Wang B., Li J., Shao Q., Guo X., Wang N., Guo Z. (2017). Crystal Structure Modification Enhanced FeNb_11_O_29_ Anodes for Lithium-Ion Batteries. ChemElectroChem.

[5] Liu M., Yang Z., Sun H., Lai C., Zhao X., Peng H., Liu T. (2016). A hybrid carbon aerogel with both aligned and interconnected pores as interlayer for high-performance lithium–sulfur batteries. Nano Res..

[6] Qu Z., Shi M., Wu H., Liu Y., Jiang J., Yan C. (2019). An efficient binder-free electrode with multiple carbonized channels wrapped by NiCo_2_O_4_ nanosheets for high-performance capacitive energy storage. J. Power Sources.

[7] Idrees M., Batool S., Kong J., Zhuang Q., Liu H., Shao Q., Lu N., Feng Y., Wujcik E.K., Gao Q. (2019). Polyborosilazane derived ceramics—Nitrogen sulfur dual doped graphene nanocomposite anode for enhanced lithium ion batteries. Electrochim. Acta.

[8] Chou S.-L., Pan Y., Wang J.-Z., Liu H.-K., Dou S.-X. (2014). Small things make a big difference: Binder effects on the performance of Li and Na batteries. Phys. Chem. Chem. Phys..

[9] Nagai A., Yoshio M., Brodd R.J., Kozawa A. (2009). Applications of Polyvinylidene Fluoride-related materials for Lithium-Ion Batteries. Lithium-Ion Batteries.

[10] Yamamoto H., Mori H., Yoshio M., Brodd R.J., Kozawa A. (2009). SBR Binder (for Negative Electrode) and ACM Binder (for Positive Electrode). Lithium-Ion Batteries.

[11] Das P.R., Komsiyska L., Osters O., Wittstock G. (2015). PEDOT: PSS as a Functional Binder for Cathodes in Lithium Ion Batteries. J. Electrochem. Soc..

[12] Shao D., Zhong H., Zhang L. (2014). Water-Soluble Conductive Composite Binder Containing PEDOT:PSS as Conduction Promoting Agent for Si Anode of Lithium-Ion Batteries. ChemElectroChem.

[13] Higgins T.M., Park S.H., King P.J., Zhang C., McEvoy N., Berner N.C., Daly D., Shmeliov A., Khan U., Duesberg G. (2016). A Commercial Conducting Polymer as Both Binder and Conductive Additive for Silicon Nanoparticle-Based Lithium-Ion Battery Negative Electrodes. ACS Nano.

[14] Crispin X., Jakobsson F.L.E., Crispin A., Grim P.C.M., Andersson P., Volodin A., van Haesendonck C., van der Auweraer M., Salaneck W.R., Berggren M. (2006). The Origin of the High Conductivity of Poly(3,4-ethylenedioxythiophene)–Poly(styrenesulfonate) (PEDOT–PSS) Plastic Electrodes. Chem. Mater..

[15] Jönsson S.K.M., Birgerson J., Crispin X., Greczynski G., Osikowicz W., van der Gon A.W.D., Salaneck W.R., Fahlman M. (2003). The effects of solvents on the morphology and sheet resistance in poly(3,4-ethylenedioxythiophene)–polystyrenesulfonic acid (PEDOT–PSS) films. Synth. Met..

[16] Wudl F., Heeger A. (1996). Self-Doped Polymers. U.S. Patent.

[17] Patil A.O., Ikenoue Y., Basescu N., Colaneri N., Chen J., Wudl F., Heeger A.J. (1987). Self-doped conducting polymers. Synth. Met..

[18] Müllen K., Reynolds J.R., Masuda T. (2013). Conjugated Polymers: A Practical Guide to Synthesis.

[19] Kuo C.-T., Chen S.-A., Hwang G.-W., Kuo H.-H. (1998). Field-effect transistor with the water-soluble self-acid-doped polyaniline thin films as semiconductor. Synth. Met..

[20] Nam S., Ko Y.-G., Hahm S.G., Park S., Seo J., Lee H., Kim H., Ree M., Kim Y. (2013). Organic nonvolatile memory transistors with self-doped polymer energy well structures. NPG Asia Mater..

[21] Chen X., Chen Z., Zhu J., Xu C., Yan W., Yao C. (2011). A novel H_2_O_2_ amperometric biosensor based on gold nanoparticles/self-doped polyaniline nanofibers. Bioelectrochemistry.

[22] Freund M.S., Deore B.A. (2007). Self-Doped Conducting Polymers.

[23] Malinauskas A. (2004). Self-doped polyanilines. J. Power Sources.

[24] Varela H., Torresi R.M., Buttry D.A. (2000). Study of charge compensation during the redox process of self-doped polyaniline in aqueous media. J. Braz. Chem. Soc..

[25] Sadoway D.R., Huang B., Trapa P.E., Soo P.P., Bannerjee P., Mayes A.M. (2001). Self-doped block copolymer electrolytes for solid-state, rechargeable lithium batteries. J. Power Sources.

[26] Liu Q., Li Z.-F., Liu Y., Zhang H., Ren Y., Sun C.-J., Lu W., Zhou Y., Stanciu L., Stach E.A. (2015). Graphene-modified nanostructured vanadium pentoxide hybrids with extraordinary electrochemical performance for Li-ion batteries. Nat. Commun..

[27] An H., Mike J., Smith K.A., Swank L., Lin Y.-H., Pesek S.L., Verduzco R., Lutkenhaus J.L. (2015). Highly Flexible Self-Assembled V_2_O_5_ Cathodes Enabled by Conducting Diblock Copolymers. Sci. Rep..

[28] An H., Li X., Chalker C., Stracke M., Verduzco R., Lutkenhaus J.L. (2016). Conducting Block Copolymer Binders for Carbon-Free Hybrid Vanadium Pentoxide Cathodes with Enhanced Performance. ACS Appl. Mater. Interfaces.

[29] An H., Li X., Smith K.A., Zhang Y., Verduzco R., Lutkenhaus J.L. (2018). Regioregularity and Molecular Weight Effects in Redox-Active Poly(3-hexylthiophene)-block-poly(ethylene oxide) Electrode Binders. ACS Appl. Energy Mater..

[30] Lee B.H., Lee J.-H., Jeong S.Y., Park S.B., Lee S.H., Lee K. (2015). Broad Work-Function Tunability of p-Type Conjugated Polyelectrolytes for Efficient Organic Solar Cells. Adv. Energy Mater..

[31] Mai C.-K., Schlitz R.A., Su G.M., Spitzer D., Wang X., Fronk S.L., Cahill D.G., Chabinyc M.L., Bazan G.C. (2014). Side-Chain Effects on the Conductivity, Morphology, and Thermoelectric Properties of Self-Doped Narrow-Band-Gap Conjugated Polyelectrolytes. J. Am. Chem. Soc..

[32] Jiang Z., Li X., Strzalka J., Sprung M., Sun T., Sandy A.R., Narayanan S., Lee D.R., Wang J. (2012). The dedicated high-resolution grazing-incidence X-ray scattering beamline 8-ID-E at the Advanced Photon Source. J. Synchrotron Radiat..

[33] Almeida E.C., Abbate M., Rosolen J.M. (2001). Formation of Li_2_O in a chemically Li-intercalated V_2_O_5_ xerogel. Solid State Ion..

[34] Wang Y., Shang H., Chou T., Cao G. (2005). Effects of Thermal Annealing on the Li+ Intercalation Properties of V_2_O_5_·nH_2_O Xerogel Films. J. Phys. Chem. B.

[35] Livage J. (1991). Vanadium pentoxide gels. Chem. Mater..

[36] Wang J., Curtis C.J., Schulz D.L., Zhang J.-G. (2004). Influences of Treatment Temperature and Water Content on Capacity and Rechargeability of V_2_O_5_ Xerogel Films. J. Electrochem. Soc..

[37] Livage J. (1998). Synthesis of polyoxovanadates via “chimie douce”. Coord. Chem. Rev..

[38] Pan A., Zhang J.-G., Nie Z., Cao G., Arey B.W., Li G., Liang S., Liu J. (2010). Facile synthesized nanorod structured vanadium pentoxide for high-rate lithium batteries. J. Mater. Chem..

[39] Ng S.-H., Patey T.J., Büchel R., Krumeich F., Wang J.-Z., Liu H.-K., Pratsinis S.E., Novák P. (2009). Flame spray-pyrolyzed vanadium oxide nanoparticles for lithium battery cathodes. Phys. Chem. Chem. Phys..

