# Supplementary material for: Self-Doped Conjugated Polymeric Binders Improve the Capacity and Mechanical Properties of V2O5 Cathodes
Source: Polymers (Basel). 2019 Apr 1;11(4):589. doi: 10.3390/polym11040589 (PMC6523139; doi:10.3390/polym11040589)
Supplement: Supplementary file 1 [file polymers-11-00589-s001.pdf]

Supporting Information for:

Self-doped conjugated polymeric binders improve the capacity and mechanical properties of V_2O_5 cathodes

Xiaoyi Li[1], Hyosung An[2], Joseph Strzalka[3], Jodie Lutkenhaus[2,4*], Rafael Verduzco[1,5+]

[1] Department of Chemical and Biomolecular Engineering, Rice University, Houston, Texas 77005, United States; xl44@rice.edu

[2] Artie McFerrin Department of Chemical Engineering, Texas A&M University, College Station, Texas 77843, United States; qcan17pp@tamu.edu

[3] X-ray Science Division, Argonne National Laboratory, Lemont, IL 60439; strzalka@anl.gov

[4] Department of Materials Science and Engineering, Texas A&M University, College Station, Texas 77843, United States.

[5] Department of Materials Science and NanoEnigneering, Rice University, Houston, Texas 77005, United States.

* Correspondence: jodie.lutkenhaus@tamu.edu

\+ Correspondence: rafaelv@rice.edu

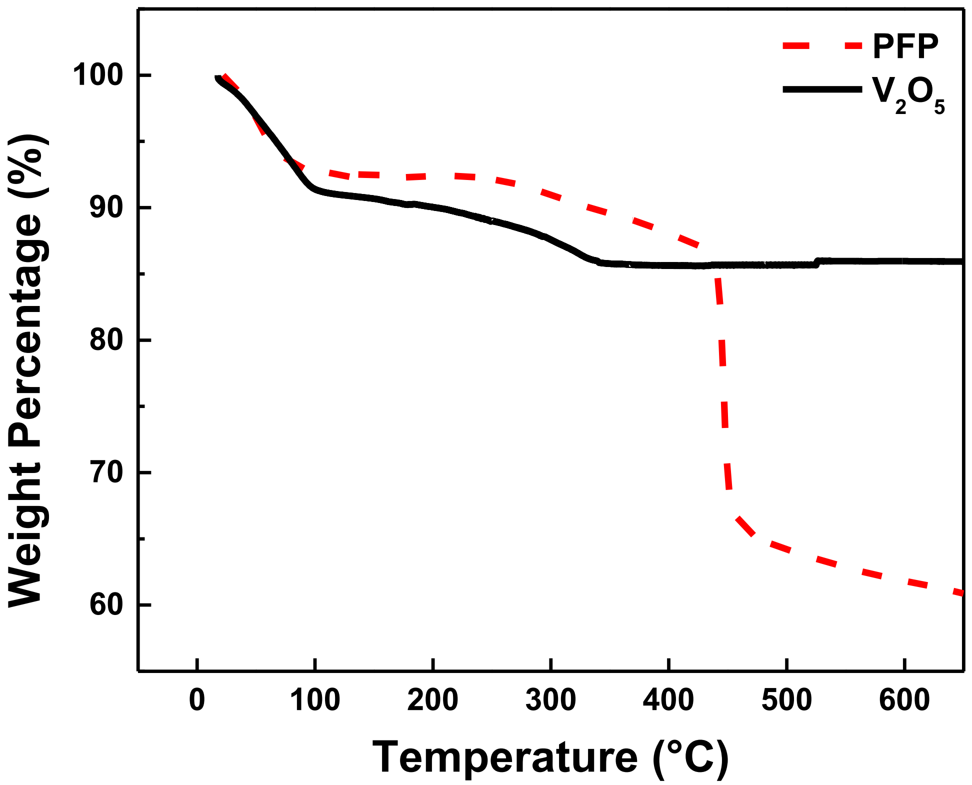

Figure S1. Thermogravimetric analysis for pure PFP, and pure V_2O_5

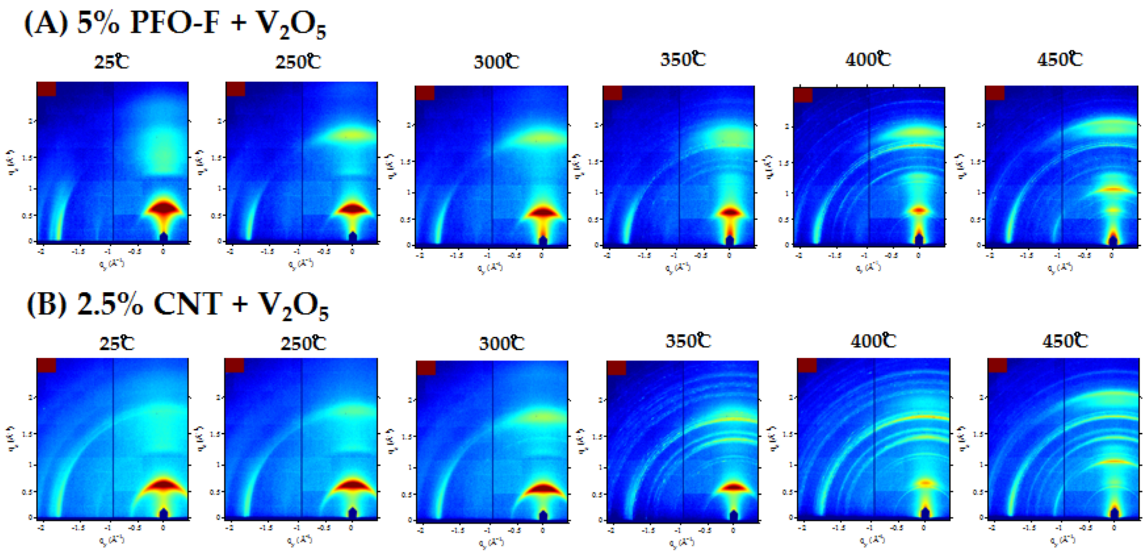

Figure S2. GIWAXS 2D images with ex-situ thermal annealing from 25°C to 450°C for V_2O_5 blended with (A) 5% PFO-F, and (B) 2.5% CNT.

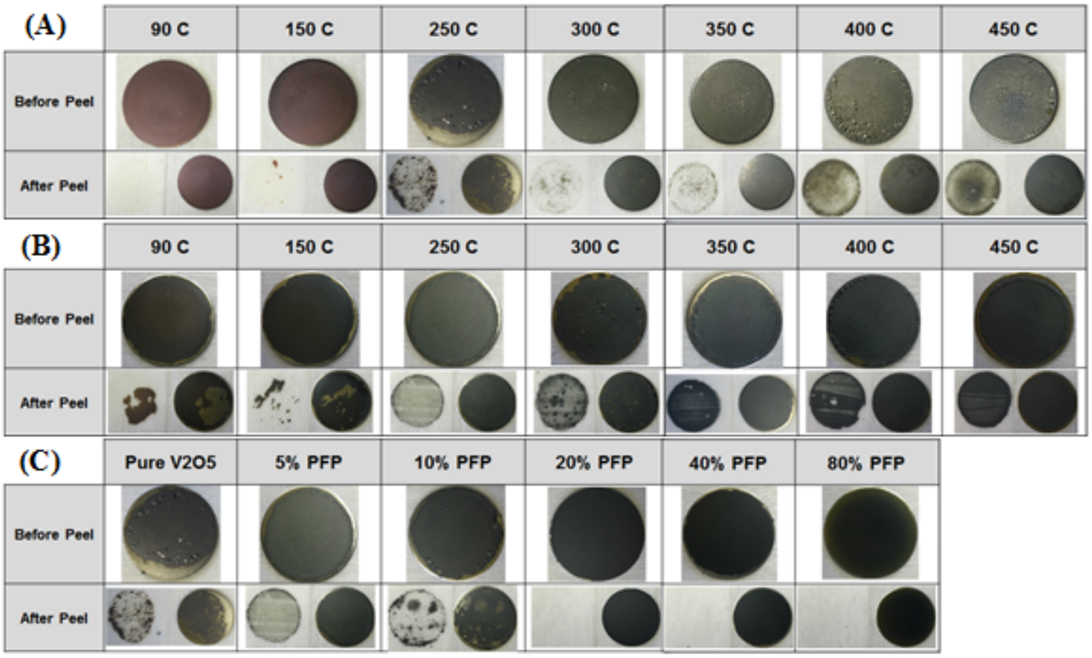

Figure S3. Images of peel tests, showing before and after peeling at different temperature ranging for 90°C to 450°C for (A) pure V_2O_5, (B) V_2O_5 + 5% PFP; (C) images of peel tests, showing before and after peeling at 250°C for different PFP polymer content from 0% to 80%.

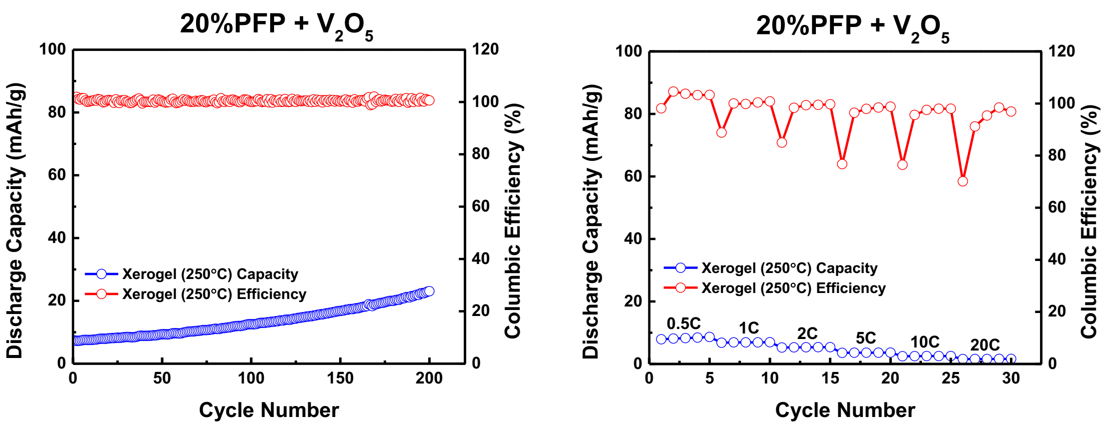

Figure S4. Galvanostatic charge-discharge tests for V_2O_5 + 20%PFP.

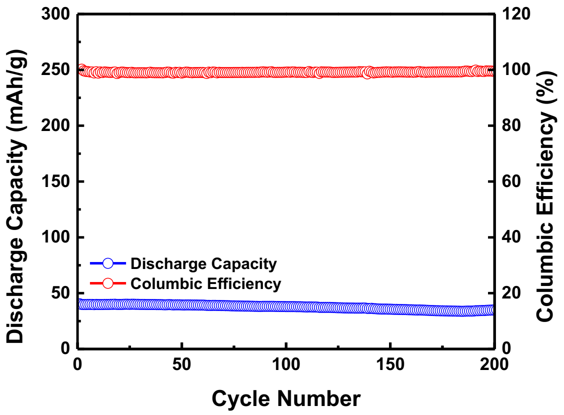

Figure S5. Galvanostatic charge-discharge tests for V_2O_5 + 10% PVDF + 10% Super-P Carbon.

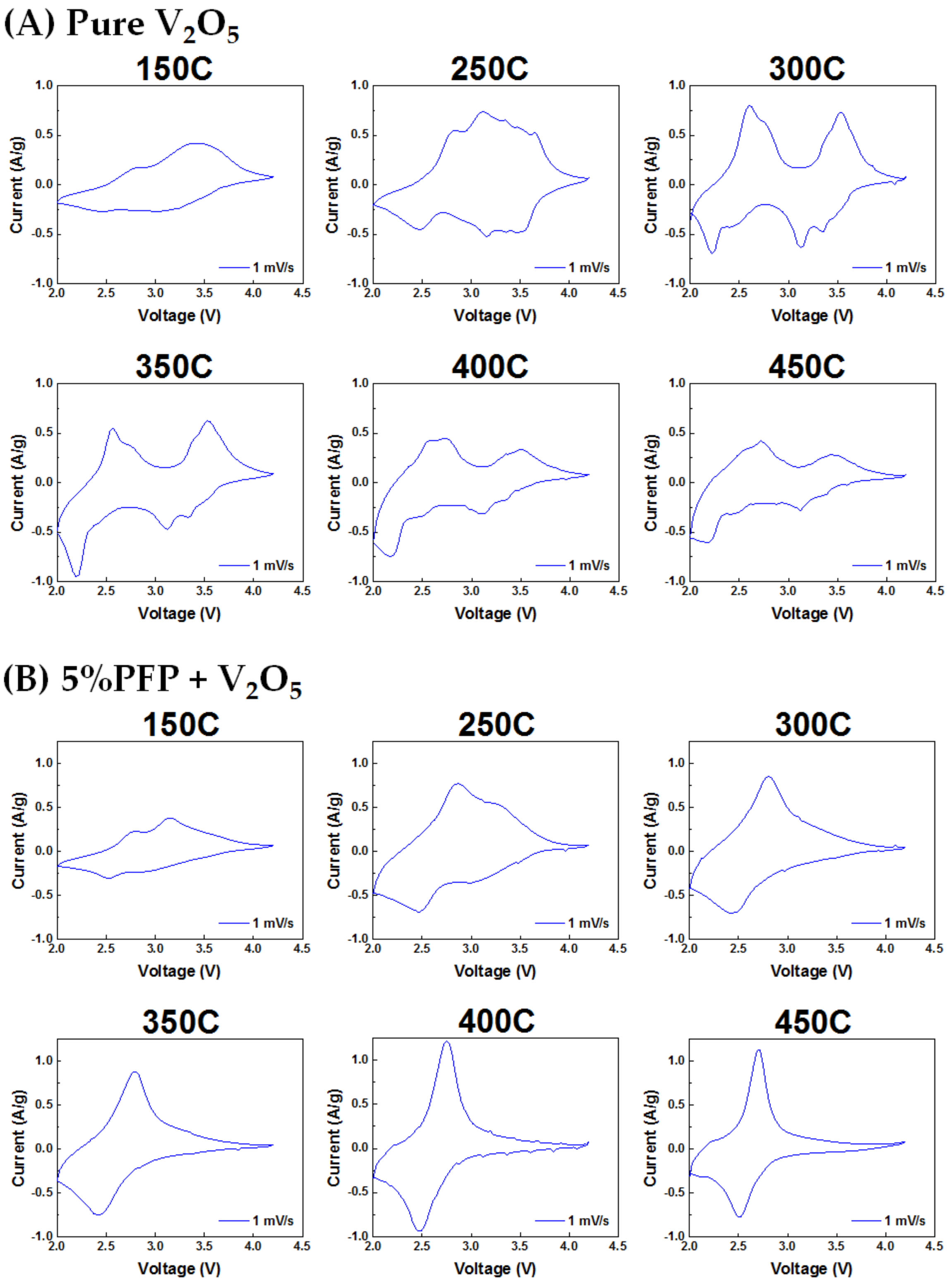

Figure S6. Cyclic Voltammetry for (a) Pure V_2O_5 and (b) 5% PFP + V_2O_5 annealed from 150°C to 450°C.
